# How does strength use relate to burnout among Chinese healthcare professionals? Exploring the mediating roles of beliefs about stress and basic psychological needs satisfaction

**DOI:** 10.1186/s12912-024-01860-w

**Published:** 2024-04-01

**Authors:** Chengzhi Bai, Jie Ma, Baoyu Bai

**Affiliations:** 1 Department of Psychology, Wuhan University, 430072 Wuhan, China; 2 Jiangsu Zhongdian Innovation Environmental Science & Technology Co., Ltd, Xian, China

**Keywords:** Strength use, Burnout, Healthcare workers, Basic psychological needs satisfaction, Beliefs about stress

## Abstract

**Background:**

The prevalence of burnout among healthcare workers remains chronically high. Former studies have indicated that strength use is a promising approach to reduce burnout. However, relatively little is known about the psychological mechanisms underlying the ability of strength use to reduce burnout, especially among healthcare workers.

**Aim:**

This study sought to examine the link between strength use and burnout in Chinese healthcare workers, and to explore the mediating roles of beliefs about stress and basic psychological needs satisfaction in that relationship.

**Methods:**

This study was conducted in two time periods, from September to October 2020 and from February to September 2022. A total of 812 healthcare workers completed a multi-section questionnaire.

**Results:**

Strength use was negatively associated with burnout and negative stress beliefs, and positively associated with positive stress beliefs, control beliefs, and basic psychological needs satisfaction. Moreover, negative stress beliefs, control beliefs, and basic psychological needs satisfaction fully mediated the relationship between strength use and burnout. Furthermore, effect contrasts revealed that the mediating effect of basic psychological needs satisfaction was stronger than that of negative stress beliefs and control beliefs.

**Conclusion:**

Our findings revealed that negative stress beliefs, control beliefs, and basic psychological needs satisfaction act as mediators in the association of strength use with burnout. Furthermore, basic psychological needs satisfaction plays a more important mediating role than negative stress beliefs and control beliefs in the strength use–burnout relationship.

## Introduction

Burnout is a common and persistent problem in the global healthcare system. It occurs when individuals experience chronic emotional and interpersonal stress as a result of their work, leading to emotional exhaustion, depersonalization, and a reduced sense of personal accomplishment over an extended period of time [1]. Recent research suggests that the incidence of burnout among healthcare professionals is increasing rapidly [2, 3]. This can have a number of serious consequences, including strained doctor-patient relationships, medical errors, substance abuse, relationship breakdowns, and suicidal thoughts [4, 5]. Therefore, it is of utmost importance to explore the factors that contribute to burnout among healthcare professionals and to develop effective intervention programs to address this issue.

In recent years, the emergence of positive psychology has offered a fresh and promising perspective on enhancing individual well-being [6, 7]. One of its most effective strategies focuses on leveraging individuals’ strengths. Previous research has demonstrated that utilizing personal strengths serves as a potent tool in mitigating burnout [8, 9]. However, our understanding of the link between strength use and burnout remains incomplete in several respects.

First, there is an apparent lack of research on the specific mechanisms by which strength use influences burnout. Identifying these mechanisms could clarify the direct pathways by which strength use affects burnout. This understanding is critical for developing targeted interventions based on strength use to effectively reduce burnout.

Second, existing studies predominantly examine Western populations, overlooking potential variability in collectivist cultures. Particularly in Asian contexts such as China, where there is a strong interdependent self-concept and emphasis on group dynamics, the relationship between strength use and burnout may differ. This cultural gap is significant because cross-cultural research shows that predictors of employee well-being are not universal, but vary according to cultural context [8]. Third, current research has focused primarily on the general population, with limited emphasis on healthcare workers. This oversight becomes particularly critical in the context of the COVID-19 pandemic. The pandemic has dramatically increased the workload of healthcare workers, posing significant risks to their physical and mental health, including increased levels of burnout. Thus, examining the role of strength use in mitigating burnout among healthcare workers during this period is both timely and necessary. Research such as that by Yip et al. [10] has already begun to uncover the transformative experiences of healthcare workers, such as junior nurses, during the pandemic, providing a starting point for further exploration.

In light of these gaps, this study aims to examine how strength use is related to burnout among Chinese healthcare workers. It seeks to uncover potential mediating mechanisms in this relationship, thereby contributing to a more nuanced understanding of how strength use can support the well-being of healthcare professionals, particularly during challenging times such as the COVID-19 pandemic.

### Strength use and burnout

Strength use refers to the degree to which individuals use their unique strengths and talents in different situations [11]. Theoretically, strength use should help reduce burnout. For example, according to the job demands-resources model [12], employees’ use of strengths at work facilitates the enhancement of personal resources, such as self-efficacy and optimism, which can buffer the negative effects of job demands on stress and enhance the positive effects of job demands on motivation. This further mitigates or prevents burnout [13]. Furthermore, empirical evidence suggests a link between using one’s strengths and experiencing burnout. For example, several studies have reported that using strengths improves several indicators of employee well-being [8, 13, 14]. Specifically, a strengths intervention study conducted by Meyers and van Woerkom [15] found that strengths use had an indirect effect on burnout via the mediator of positive affect, but no direct effect. Thus, based on the existing theoretical and empirical evidence, we formulated the following hypotheses:

#### Hypothesis 1

Strengths use is negatively related to burnout in healthcare workers.

### Strength use, beliefs about stress and burnout

Beliefs about stress may serve as a mediator in the relationship of strength use with burnout. To our knowledge, no empirical studies have directly examined the mediating effect of beliefs about stress. Beliefs about stress refer to people’s beliefs about the effects of stress on their health and performance (e.g., positive or negative) and their perceptions of whether they can effectively manage stress. The concept of beliefs about stress encompasses three types of beliefs, as follows: Positive stress beliefs are beliefs that stress is positive and beneficial to health and performance; Negative stress beliefs are beliefs that stress is negative and detrimental to health and performance; Control beliefs are beliefs that one can effectively control stress [16]. Previous research has shown that a number of stress-related health and behavioral outcomes reflect people’s lay beliefs and expectations about these outcomes [16, 17]. The positive activity model [18] supports the idea that beliefs about stress may mediate the relationship between strength use and burnout. This model explains how positive activities can enhance well-being and provides insight into why this occurs, suggesting that positive activities such as strength use may enhance well-being through positive thoughts, in which case the individual perceives stress as positive and controllable rather than negative.

Some evidence appears to support this mediation model. Although no previous studies have directly demonstrated the relationship, the existing evidence seems to suggest that strength use is related to beliefs about stress. In theoretical arguments, Bakker and Woerkom [12] have argued that strength use may promote individuals’ personal resources, such as optimism and self-efficacy, which allow them to feel in control of their external environment and further strengthen control beliefs. Moreover, several studies have shown that the use of strengths can enhance positive emotions [19, 20]. According to the broaden and build theory [21], positive emotions can serve as a resource that helps individuals cope with stressful events or threats and succeed in a variety of situations, and therefore may be beneficial for individuals’ beliefs about stress. In other words, strength use may contribute to one’s positive stress beliefs and control beliefs, while reducing negative stress beliefs. Therefore, we can infer that strength use may be beneficial for individuals’ beliefs about stress. More specifically, strength use may strengthen positive stress beliefs, strengthen control stress beliefs, and weaken negative stress beliefs.

Moreover, empirical studies have identified a connection between beliefs about stress and burnout. For example, stress mindset describes the extent to which people hold enhanced or diminished beliefs about the consequences of stress, much like positive and negative stress beliefs [22]; studies have shown that stress mindset is significantly associated with burnout in students [23], community members [24], and employees [25]. Considering the relationships of beliefs about stress with strength use and burnout, we made the following hypothesis:

#### Hypothesis 2

Beliefs about stress mediate the link of strength use with burnout in healthcare workers.

### Strength use, basic psychological needs satisfaction and burnout

Basic psychological needs satisfaction (BPNS) may also play a mediating role in the association between strength use and burnout. Theoretically, the positive activity model [18] suggests that BPNS is one of the four mediators between positive activities, such as strength use, and well-being. Because burnout is an important indicator of well-being, especially among healthcare workers, we examined the relationship between strength use and burnout from the perspective of BPNS. BPNS is a key component of self-determination theory [26], which suggests that people have three basic psychological needs, namely autonomy, competence, and relatedness. Research has shown that individuals who frequently use their strengths tend to have higher levels of BPNS. For example, Bai et al. [27] reported a positive relationship between strength use and BPNS using cross-sectional surveys. In addition, previous research has shown that BPNS is associated with burnout in various populations, such as athletes [28], students [29], and teachers [30]. Taken together, these findings suggest a strong relationship between strength use, BPNS, and burnout. Therefore, we hypothesized the following:

#### Hypothesis 3

BPNS mediates the link of strength use with burnout.

### Current research

In this study, our aim was to examine the relationship between strength use and burnout in Chinese healthcare workers. In addition, we sought to examine the mediating roles of beliefs about stress and BPNS in the relationship between strength use and burnout. Based on the theoretical and empirical evidence presented above, we formulated the following three hypotheses:

(1) The use of strengths is inversely related to burnout among Chinese healthcare workers.

(2) Beliefs about stress serve as a mediator in the strength use-burnout relationship among healthcare workers in China.

(3) BPNS functions as a mediator in the relationship between strength use and burnout among Chinese healthcare workers.

## Methods

### Study design and participants

This study employed a repeated cross-sectional design, conducted in two separate phases using convenience sampling, to comprehensively assess the impact of the COVID-19 pandemic on healthcare workers in China. Data collection was facilitated through an online questionnaire platform (https://www.wjx.cn/). The initial phase of the study took place from September to October 2020, followed by the second phase which extended from February to September 2022. This two-phase approach enabled a thorough exploration of the evolving effects of the pandemic on this critical workforce over time.

### Phase 1: Workers from the centers for disease control and prevention (CDC) in Hubei Province (September to October 2020)

The study involved 367 CDC workers from major cities in Hubei Province, including Wuhan, which was the initial epicenter of the COVID-19 outbreak in China. This group provided invaluable insights into the early stages of the pandemic and the initial public health response. Importantly, data from these participants were previously used in another study [9] that focused on the interplay between strength use and burnout from a psychological capital perspective. The current research extends this by examining the same relationship through the lens of BPNS and stress beliefs, providing a novel and complementary understanding of the dynamics involved. The primary goal of this phase was to assess the immediate aftermath of the easing of the lockdown, capitalizing on the direct experience of CDC staff in frontline pandemic management and reflecting broader trends in public health response.

### Phase 2: Nurses in three Chinese provinces (February to September 2022)

In the second phase, the study expanded its geographic and professional scope to include 578 nurses from three different Chinese provinces. Conducted in 2022, this phase aimed to capture the evolving challenges and stressors nurses faced amidst ongoing pandemic waves and policy changes. This phase’s focus on nurses in diverse clinical settings provided a contrasting perspective to the experiences of CDC workers, enriching the longitudinal analysis of healthcare workers’ experiences at different stages of the pandemic. By integrating findings from both phases, the study provides a comprehensive view of the impact of the pandemic on health care workers and enhances our understanding of the long-term effects and resilience strategies in the face of COVID-19.

### Inclusion and exclusion criteria

For this study, the inclusion criteria were designed to specifically target CDC workers in Hubei Province and nurses in three Chinese provinces who played active roles in patient care and pandemic management during the COVID-19 crisis within the study periods. This included professionals directly engaged in tasks such as patient treatment, testing, and public health initiatives related to COVID-19. Conversely, our exclusion criteria encompassed individuals not directly participating in COVID-19 response efforts, those absent or on leave during data collection, and participants submitting incomplete or inconsistent survey responses. This approach was crucial to ensure that our sample accurately and comprehensively represented the experiences and perspectives of frontline healthcare workers during the pandemic, thereby enhancing the validity of our findings.

## Data collection

### Instruments

#### Strengths use

Strengths use was measured by the 14-item Strengths Use Scale [11], which measures the level of strength use in individuals. Items are scored on a 7-point Likert-type scale ranging from “1 = completely disagree” to “7 = completely agree”. Higher mean scores represent more strength use. This scale has exhibited outstanding reliability and validity in the context of Chinese culture [27, 31, 32]. In the present study, the Cronbach’s alpha was 0.975.

#### Beliefs about stress

The Beliefs about Stress Scale (BASS) was developed by Laferton et al. [16]. The 15-item BASS has three dimensions, including negative stress beliefs (BASS-N; 8 items), positive stress beliefs (BASS-P; 4 items), and control beliefs (BASS-C; 3 items). Each item is scored on a 5-point scale ranging from “1 = completely disagree” to “5 = completely agree”. The overall mean score was calculated for each dimension, whereby a higher score indicated a stronger belief. Former research has indicated that this scale exhibits strong reliability and validity among Chinese college students [33]. In this study, the alpha coefficients of the three BASS-N, BASS-P, and BASS-C dimensions were 0.828, 0.900, and 0.803, respectively.

#### Basic psychological needs satisfaction

In this research, we used the abbreviated version of the Basic Psychological Needs Satisfaction (BPNS) scale developed by Sheldon and Niemiec [34]. This instrument, specifically designed to measure the satisfaction of basic psychological needs, consists of nine items across three critical dimensions: autonomy, competence, and relatedness satisfaction. Each item is scored on a comprehensive 7-point Likert scale ranging from “not at all” to “completely” allowing for a nuanced assessment of need satisfaction. Higher scores on this scale indicate greater satisfaction with these intrinsic needs. Of note, the scale has been shown to be robust and valid in the Chinese context [35, 36], making it particularly appropriate for the demographics of this study. The reliability of the scale, as evidenced by a Cronbach’s alpha coefficient of 0.770 in our study, underscores its consistency in measuring the intended constructs.

#### Burnout

The Chinese Maslasch Burnout Inventory, translated and revised by Li et al. [37], was used to assess burnout in the study participants. We were authorized to use the Chinese scale before we started our study. The questionnaire has 15 items, which cover the three dimensions of emotional exhaustion, depersonalization, and reduced professional efficacy. Items are scored on a 7-point scale ranging from 0 (never) to 6 (every day), and each dimension was scored as the mean of the corresponding item. Higher levels of burnout are indicated by lower personal fulfillment dimension scores and higher emotional exhaustion and dehumanization dimension scores. The Cronbach’s alpha coefficient in this study was 0.910.

### Ethical considerations

Ethical approval for this study was obtained from the Ethics Committee of the Department of Psychology, School of Philosophy, Wuhan University (approval number: 2,020,071,601). Informed consent was obtained from all participants to ensure that they were fully aware of the aims and procedures of the study. We strictly protected the privacy and confidentiality of participants by anonymizing all data and limiting access to the research team. Measures were taken to ensure that individual responses could not be traced back to participants, thereby maintaining their anonymity throughout the study.

### Data analysis

Our analysis began with descriptive statistics, which provided a snapshot of the core trends and dispersion of the data set. Correlation analysis was then performed to unravel the relationships between variables. As we delved into the predictive intricacies of our dataset, we embarked on multiple regression analyses. This step was critical in identifying the unique impact of different predictors on the outcomes of interest. In addition, we employed PROCESS Macro V3.4.1 to uncover the mediating influence of certain factors within our framework. This analysis phase was instrumental in deconstructing the nuanced indirect effects that our independent variables exerted on the dependent variables through one or more mediators. The adoption of 5000 bootstrap samples for this phase, coupled with the formulation of 95% confidence intervals, strengthened the robustness and credibility of our inferences regarding these mediated pathways. Specifically, the significance of mediation is confirmed when the 95% confidence interval (CI) does not intersect zero.

## Results

### Descriptive statistics and correlation analysis

Out of the 945 questionnaires collected for the study, 133 were excluded from analysis due to invalid responses. For the data analysis, data from a final total of 812 respondents were selected from this sample. Among these, 351 were Centers for Disease Control and Prevention staff and 461 were nurses; 649 (79.9.1%) were women and 163 were men (20.1%). The age distribution was as follows: 152 (18.72%) were aged 21–30 years, 281 (34.61%) were aged 31–40 years, 229 (28.2%) were aged 41–50 years, and 150 (18.47%) were aged 50 years or older.

Table 1 shows the correlations of strength use, beliefs about stress, BPNS, and burnout. Unsurprisingly, all variables were significantly correlated with each other (*p* < 0.001).

**Table 1:** Descriptive statistics and correlations among the key variables

Variable	M [SD]	Correlation					
		1	2	3	4	5	6
1. Strength use	4.55[1.09]	—					
2. BASS-P	4.29[1.17]	0.538***	—				
3. BASS-N	4.54 [0.92]	− 0.214***	− 0.370***	—			
4. BASS-C	4.62[0.98]	0.596***	0.652***	− 0.235***	—		
5. BPNS	4.53[0.77]	0.591***	0.360***	− 0.260***	0.504***	—	
6. Burnout	36.05[16.25]	− 0.429***	− 0.385***	0.425***	− 0.429***	− 0.560***	—

Note. ***p < 0.001. Abbreviation: BASS = Beliefs About Stress Scale, BASS-P = Positive stress beliefs, BASS-N = Negative stress beliefs, BASS-C = Control beliefs, BPNS = Basic psychological needs satisfaction, M [SD] = Mean [Standard Deviation]

### Multiple mediation analysis

We performed a multiple mediation analysis in PROCESS [38] to explore the simultaneous, independent contributions of positive stress beliefs, negative stress beliefs, control beliefs, and BPNS to the relationship of strength use with burnout. Our findings revealed that the entire model accounted for 44.7% of the variation in burnout, and the overall explanation of burnout by strength use was 23%. Moreover, as shown in Table 2, the 95% CIs for negative stress beliefs (estimate = − 0.054, 95% CI = [-0.079, − 0.03]), control beliefs (estimate = − 0.063, 95% CI = [-0.115, − 0.012]), and BPNS (estimate = − 0.22, 95% CI = [-0.274, − 0.168]) did not contain zero; meanwhile, the 95% CIs for positive stress beliefs (estimate = − 0.027, CI = [-0.076, 0.018]) crossed zero, thus suggesting that strength use affects burnout indirectly through negative stress beliefs, control beliefs, and BPNS, rather than positive stress beliefs. Additionally, the total effect of strength use on burnout was significant (estimate = − 0.416, 95% CI = [-0.476, − 0.355]). After controlling for mediators, the direct association between strength use and burnout became non-significant (estimate = − 0.051, 95% CI = [-0.124, 0.022]), which suggested that negative stress beliefs, control beliefs, and BPNS fully mediated this association.

**Fig. 1:**
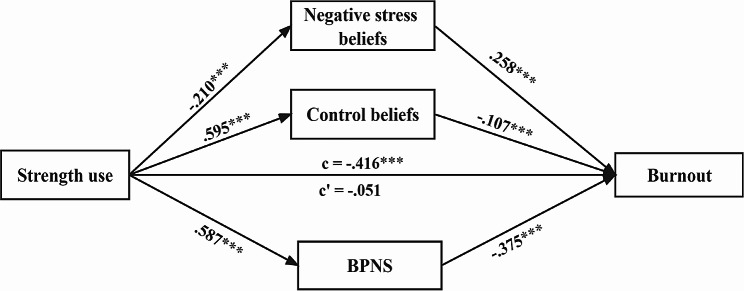
The mediating effects of negative stress beliefs, control beliefs, and basic psychological needs satisfaction (BPNS) between strength use and burnout. Note: ***p < 0.001

Comparative tests were conducted to determine whether negative stress beliefs, control beliefs, or BPNS was a stronger contributor to the link between strength use and burnout. As presented in Table 2, there was a significant difference between the mediating effect of BPNS and that of negative stress beliefs and control beliefs, because the 95% CI of the mediating effect of BPNS minus that of negative stress beliefs (estimate = 0.166, 95% CI = [0.106, 0.225]) and control beliefs (estimate = 0.156, 95% CI = [0.074, 0.260]) did not include zero. Moreover, the mediating effect of negative stress beliefs was not significantly different from that of control beliefs, because the 95% CI of the mediating effect of negative stress beliefs minus that of control beliefs contained zero (estimate = 0.009, 95% CI = [-0.045, 0.065]). These results imply that BPNS played the strongest mediating role in the linkage of strength use with burnout, and negative stress beliefs and control beliefs played equally important mediating roles in this relationship (see Fig. 1).

**Table 2:** The Effects and 95% Confidence Intervals

Model pathways	Estimated	95% CI	
		Lower	Upper
Direct effect			
Strength use→burnout	-0.051	-0.124	0.022
Indirect effect			
Strength use→burnout	-0.365^a^	-0.430	-0.299
Strength use→ BASS-P→ burnout	-0.027	-0.076	0.018
Strength use→ BASS-N→ burnout	-0.054^a^	-0.079	-0.030
Strength use→ BASS-C→ burnout	-0.063^a^	-0.115	-0.012
Strength use→ BPNS→ burnout	-0.220^a^	-0.274	-0.168
IndEff [BASS-N] minus IndEff [BASS-C]	0.009	-0.045	0.065
IndEff [BASS-N] minus IndEff [BPNS]	0.166^a^	0.106	0.225
IndEff [BASS-C] minus IndEff [BPNS]	0.156^a^	0.074	0.240

^a^ Empirical 95% confidence interval does not overlap with zero. IndEff = Indirect effect. BASS = Beliefs About Stress Scale, BASS-P = Positive stress beliefs, BASS-N = Negative stress beliefs, BASS-C = Control beliefs, BPNS = Basic psychological needs satisfaction

## Discussion

Our study investigated the correlation between using personal strengths and experiencing burnout, as well as the mediating role of beliefs about stress and BPNS in Chinese healthcare workers from the perspective of the positive activities model. Our results suggested that the use of strengths can affect burnout by the mediating effects of negative stress beliefs, control beliefs, and BPNS. The main contributions of our work will now be discussed.

First, our correlational results showed that strength use was inversely related to burnout, consistent with previous studies [8, 9]. These collective findings not only underscore the importance of strength use as an important protective factor against burnout, but also reinforces the universal applicability of strengths-based interventions in diverse cultural contexts and challenging situations, such as the COVID-19 pandemic. Moreover, our research deepens the ecological validity of the relationship between strength use and burnout by situating it within the distinctive and challenging context of the COVID-19 pandemic in China. The pandemic has significantly exacerbated stress and burnout levels, particularly among healthcare workers, making this scenario particularly relevant for examining such relationships. By focusing on healthcare workers during this pivotal period, our research not only examines this relationship in an environment of heightened relevance, but also provides practical recommendations for the management of health authorities. Our findings advocate the strategic application of strengths-based approaches to mitigate burnout among health workers, a key consideration in managing pandemic responses and overall health system resilience. This approach, which emphasizes the development and use of health workers’ strengths, is emerging as an effective strategy for mitigating burnout [8–15], thereby contributing to both individual well-being and the stability of health systems under duress.

Second, this study contributes to our understanding of the antecedents of beliefs about stress, an area that has been less explored compared to the consequences of these beliefs. While most research in this area has focused on how beliefs about stress influence stress-related behaviors and health outcomes [16, 17], identifying the factors that shape these beliefs is critical to developing effective stress management strategies. Our study uniquely demonstrates that the use of personal strengths plays an important role in fostering positive beliefs about stress control and reducing negative perceptions of stress. This finding is particularly relevant because it suggests a proactive approach to stress management that emphasizes the development of personal strengths. By increasing our knowledge of what leads individuals to view stress in a positive or negative light, we can better equip them to manage stress effectively, potentially leading to improved health and performance outcomes [17]. Future research should continue to explore a broader range of antecedents to these beliefs and further enrich our understanding of the dynamics of stress management.

Third, the central finding of our study is that negative stress beliefs, control beliefs, and BPNS fully mediate the relationship between personal strength use and burnout. This intricate interplay reveals that stress beliefs and BPNS independently mediate the impact of strength use on burnout, significantly enriching our understanding of the dynamics between strength use and burnout and offering profound theoretical implications. Uniquely, our study is the first to demonstrate that the relationship between strength use and burnout is mediated by a change in stress beliefs. We found that the use of personal strengths reduces negative stress beliefs and strengthens control beliefs, which together contribute to a reduction in burnout. This finding provides robust empirical support for the positive activity model [18], which suggests that positive psychological activities, such as the use of strengths, enhance well-being through adaptive cognitive processes, including the modification of stress beliefs. Our research contributes a novel perspective to this model by explicitly detailing the influence of strength use on stress beliefs, thereby facilitating positive psychological outcomes in high-stress environments. This finding not only expands our theoretical understanding of how strength use affects burnout, but also provides practical, evidence-based strategies for using individual strengths to effectively mitigate workplace stress and burnout.

In addition, our research uniquely uncovers the mediating role of BPNS in the strength use-burnout relationship. By demonstrating that strength use can promote positive outcomes through BPNS, we provide direct evidence supporting a key tenet of the positive activity model. This finding is also partially consistent with recent research suggesting that higher strength use correlates with lower depressive symptoms in nurses, mediated by increased BPNS [27]. Taken together, these findings underscore the importance of both strength use and the satisfaction of basic psychological needs in enhancing the well-being of healthcare workers, particularly in challenging contexts such as the current pandemic.

Another significant finding was that BPNS acted as a stronger mediator of the strength use-burnout relationship than negative stress beliefs and control beliefs. This finding extends the positive activity model [18], which proposes that positive activities lead to positive outcomes through four mediating variables, but does not specify whether the same positive activity triggers all four mediating variables simultaneously. Furthermore, to our knowledge, no previous study has examined whether two or more of these four mediating variables occur simultaneously during the same positive activity, or which of these variables has the strongest mediating role. Our study suggests that the same positive activity, strength use, involves two variables (positive beliefs and BPNS) that in turn lead to positive outcomes, and that the BPNS model is stronger. Future research could examine the other two mediating variables to determine whether the same positive activity involves all four mediating variables simultaneously, and further test which mediating pathway is more significant. This could have important practical implications for the design of intervention programs based on this theoretical model.

### Recommendations for future research

This study has several limitations that should be noted. First, despite the relatively large sample size, all participants were recruited from China. Future studies should include samples from different cultures and different groups to expand the generalizability of our findings. Second, as a cross-sectional study, our results cannot establish causality or sufficiently confirm mediating mechanisms. Future longitudinal or experimental studies should be conducted to further investigate the directionality of the relationships among personal strengths use, stress beliefs, BPNS, and burnout. Finally, the self-report method we used to measure the variables may have been affected by subjective response bias. Future studies should employ multiple assessment methods to minimize this potential bias.

## Conclusion

In this study, we investigated how beliefs about stress and BPNS influence the relationship between strength use and burnout among Chinese healthcare workers. Our findings reveal that negative stress beliefs, control beliefs, and BPNS act as mediators in the connection between strength use and burnout in Chinese nurses. Notably, BPNS emerged as the most potent mediator, exerting a stronger influence than both negative stress beliefs and control beliefs.

### Implications for management

The results of our research have the following implications for healthcare workers and managers. First, when organizations are faced with problems such as burnout, they should encourage healthcare workers to use strengths rather than minimize weaknesses. Strengths interventions have been shown to be a promising way to improve employee well-being and reduce psychological distress [6, 11]. Second, when objective stressors cannot be reduced or avoided, changing healthcare workers’ beliefs about stress is an effective alternative way to alleviate burnout, and interventions could be developed that rely on this strategy to enhance positive stress beliefs and control beliefs. Third, it is important to meet the BPNS of healthcare workers. To do this, it may be necessary to develop interventions and strategies that meet the basic psychological needs of healthcare workers according to their work and life characteristics.

## Data Availability

The datasets generated during and/or analyzed during the present study are available from the corresponding author on reasonable request.
